# Low-Grade Fibromyxoid Sarcoma: Incidence, Treatment Strategy of Metastases, and Clinical Significance of the *FUS* Gene

**DOI:** 10.1155/2013/256280

**Published:** 2013-05-30

**Authors:** Katja Maretty-Nielsen, Steen Baerentzen, Johnny Keller, Heidi Buvarp Dyrop, Akmal Safwat

**Affiliations:** ^1^Sarcoma Centre of Aarhus University Hospital, Denmark; ^2^Department of Experimental Clinical Oncology, Aarhus University Hospital, Noerrebrogade 44, 8000 Aarhus C, Denmark; ^3^Department of Pathology, Aarhus University Hospital, Noerrebrogade 44, 8000 Aarhus C, Denmark; ^4^Department of Orthopaedic Surgery E5, Aarhus University Hospital, Noerrebrogade 44, 8000 Aarhus C, Denmark; ^5^Department of Oncology, Aarhus University Hospital, Noerrebrogade 44, 8000 Aarhus C, Denmark

## Abstract

*Aim*. The aim of this study was to assess the incidence of low-grade fibromyxoid sarcoma (LGFMS), present treatment results of metastatic LGFMS, and investigate the clinical significance of the *FUS* gene rearrangement. *Methods*. This study included 14 consecutive LGFMS patients treated at the Aarhus Sarcoma Centre in 1979–2010. Fluorescent in situ hybridization (FISH) analysis for *FUS* break-apart was performed for all patients. *Results*. The incidence of LGFMS was 0.18 per million, representing 0.6% of all soft tissue sarcomas. Four patients needed multiple biopsies/resections before the correct diagnosis was made. Four patients experienced local recurrence, and three patients developed metastases. The treatment of metastatic LGFMS varied from multiagent chemotherapy to repeated, selective surgery of operable metastases. The best response to chemotherapy was short-term stabilization of disease progression, seen with Trabectedin. The prevalence of the *FUS* break-apart was 21.4%. We found no significant difference in clinical characteristics and outcomes in correlation with the *FUS* break-apart. *Conclusion*. LGFMS is a rare disease with multiple challenges. The *FUS* break-apart was not associated with local recurrence or metastases in our study. To date the only treatment resulting in disease-free periods is surgery; however further investigation into the management of metastatic LGFMS is necessary.

## 1. Introduction

Low-grade fibromyxoid sarcoma (LGFMS) is characterized by its relatively benign histological appearance with spindle cells in a whorling pattern, as well as collagenized and myxoid areas [1, 2]. The heterogeneous histological appearance makes the diagnosis challenging. In spite of the low-grade and benign histological appearance, early studies of retrospectively diagnosed LGFMS have shown a distinct biological behaviour, with a relatively high and atypical metastasizing potential, making the correct diagnosis of LGFMS important [1, 2]. Likely due to the difficulties in diagnosing LGFMS, the incidence has not been previously described.

LGFMS raises many clinical challenges. The metastatic potential raises the question whether surgery alone, which is standard treatment for other low-grade soft tissue sarcomas, is enough. The atypical metastatic potential, with atypical sites of metastases and relapses long after primary treatment, affects the choice of the best imaging method as well as the optimal duration of followup. The lack of sensitivity to radiotherapy and chemotherapy makes it important to identify patients with high risk of metastasis and obtain more information on the course of their disease and treatment, in order to evaluate and improve the treatment strategy.

Previous studies have shown that the chromosomal translocation t(7;16)(q32-34; p11), producing a *FUS/GREB3L2* fusion gene, is characteristic for the LGFMS; however the clinical significance of the translocation remains uncertain [3, 4].

The aim of this study was to assess the incidence of LGFMS, present treatment results of metastatic LGFMS, and investigate the clinical significance of the *FUS* gene rearrangement. 

## 2. Patients and Methods

LGFMS patients were identified using a validated, population-based Danish clinical database, the Aarhus Sarcoma Registry (ASR). Since 1979, the treatment of patients with sarcoma in western Denmark has been centered at the Sarcoma Centre of Aarhus University Hospital, which covers a population of approximately 2.5 million. All patients treated at the centre since 1979 have been registered in the ASR. The ASR collects basic patient data, specific data on tumor characteristics and treatment, and data on followup, local recurrence, and distant metastasis. Data in the ASR have been systematically validated and were found to be population-based, with a completeness of 85.3%, on an individual level [5].

**Figure 1:**
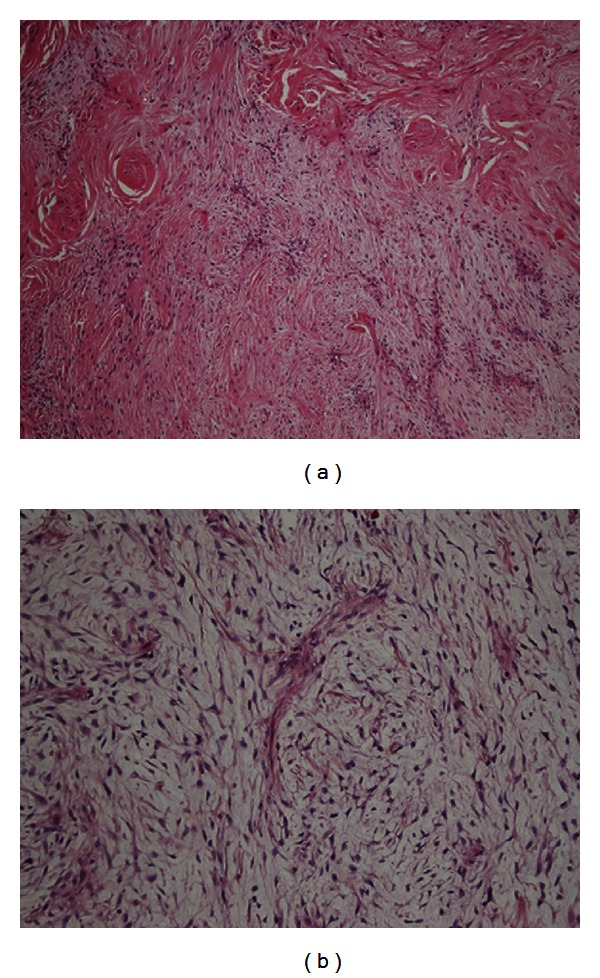
Low-grade fibromyxoid sarcoma with (a) fibrous parts (including hints of collagenous rosettes) on the upper left and myxoid parts opposite (case 1) H&E ×100 and (b) myxoid parts with classic whorling spindle cells and curvilinear thin vessels (case 14) H&E ×200.

 The inclusion criteria for this study were patients registered in the ASR between 1979 and 2010 with a low-grade fibrosarcoma, resulting in a total of 30 patients. Tissue sections stained with hematoxylin and eosin from the 30 patients were reviewed by a pathologist with expertise in sarcomas, and based on the characteristic histopathology 14 cases of LGFMS were diagnosed retrospectively (Figures 1(a) and 1(b)).

 The incidence of LGFMS in western Denmark was assessed as an incidence rate (IR) per million inhabitants in the period of 1979 to 2010. Data on the population size of western Denmark in this period was attained from StatBank Denmark, a database containing detailed statistical information on the Danish society, including number of citizens per calendar year [6]. The proportion of LGFMS was assessed in relation to all soft tissue sarcomas, including aggressive fibromatosis, in the ASR.

 The medical files of the 14 patients were retrieved from both the Department of Orthopedic Surgery and the Department of Oncology, and the course of the disease was reviewed.

 Fluorescent in situ hybridization (FISH) analysis for *FUS* break-apart (indicating the *FUS/CREBL3L2* translocation) was performed on formalin-fixed, paraffin-embedded tissue sections from the 14 patients, at the Department of Pathology, Aarhus University Hospital, Denmark, using the following kits: Abbott: Vysis *FUS* Break Apart FISH Probe Kdit, code 3N58-20 and Dako: Histology FISH Accessory Kit, code K5799. The tissue sections were scored by evaluating 200 nonoverlapping tumor nuclei per sample, and rearrangement in more than 10% of the cells was considered a positive result. Three of the cases had previously been analyzed at the Department of Pathology, Copenhagen University Hospital, Denmark; however the analyses were repeated to ensure uniform assessment.

 Patients who tested positive for the *FUS *break-apart were compared with patients who tested negative according to age, sex, local recurrence, metastases, and tumor size using *t*-test, chi-squared test, and Wilcoxon-Mann-Whitney test, respectively. All tests were two-sided, and a *P* value of <0.05 was considered significant. Analyses were performed using Stata statistical software, version 11.2.

## 3. Results

### 3.1. LGFMS Population

Overall, 14 cases of LGFMS were identified in the Aarhus Sarcoma Registry, corresponding to a crude IR of 0.18 per million (95% CI 0.10–0.30) in western Denmark from 1979 to 2010 and 0.6% (95% CI 0.4–1.1) of diagnosed soft tissue sarcomas, including aggressive fibromatosis.  Table 1 presents clinical information on each patient. There were five males (36%) and nine females (64%), and the median age at diagnosis was 36 years (range 8–65 years). The primary tumor was located in the proximal extremity or trunk in 11 of the patients and subfascial in 13 of the patients. The median tumor size at diagnosis was 4.5 cm (range 2–26 cm).

### 3.2. Diagnosis of LGFMS

The standard diagnostic program at the Aarhus Sarcoma Centre includes a clinical examination, magnetic resonance imaging (MRI) scan of the tumor area, chest X-ray or computerized tomography (CT) scan, and core-needle or incisional biopsy.

When reviewing the medical files of the 14 patients, we found that making the correct histopathological diagnosis of LGFMS was difficult in five cases. Case 1 underwent repeated biopsies and resections of primary tumor, local recurrences, and metastasis over a period of 11 years where the diagnosis varied from a benign fibromatous tumor to aggressive fibromatosis, neurofibroma, and neurofibromatosis, before the diagnosis of LGFMS was made. In cases 9 and 12 core-needle biopsies showed benign lesions, and the LFGMS diagnosis was only made at the final resection. Cases 13 and 14 underwent multiple core-needle and incisional biopsies from the primary tumor and/or metastases, all inconclusive and unable to determine whether the lesions were benign or malignant, before the diagnosis of LGFMS was made.

**Figure 2:**
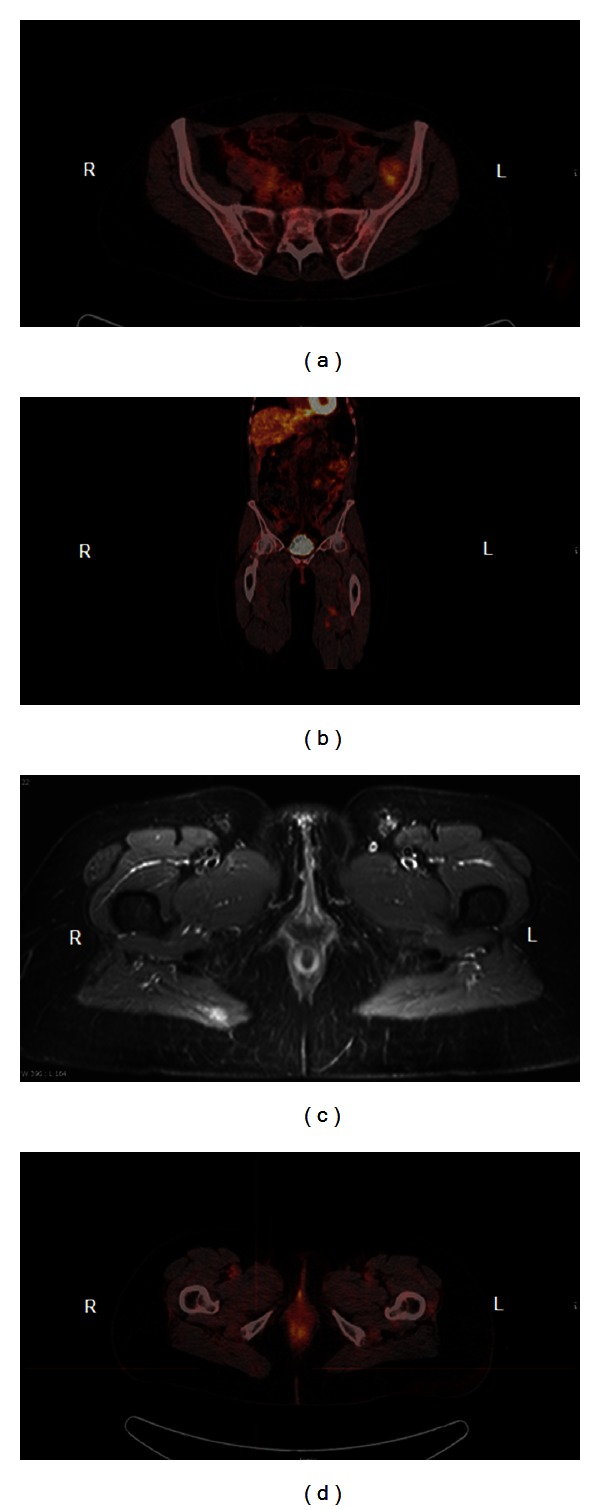
Imaging of the pelvic area in case 14. PET-CT scan showing three positive lesions on the left side: one in the pelvic area (a) and two in the thigh (b). MRI scan showing an additional lesion in the gluteal muscle on the right side (c), not positive on the PET-CT scan (d).

Positron emission tomography-computerized tomography (PET-CT) was used as a part of the diagnostic program in one case (case 14). Initially the patient presented with tumors in the right lung and mediastinum, diagnosed on a CT scan. A PET-CT showed only slightly positive lesions in the right lung and no positive lesions elsewhere. However only three months later a CT scan showed growth of a gluteal tumor, compared to the previous PET-CT, which after removal was assumed to be the primary tumor. To determine the status of the disease a new PET-CT scan was carried out a year later, revealing three new lesions on the left side: one in the pelvic area (Figure 2(a)) and two in the thigh (Figure 2(b)). An MRI scan was conducted preoperatively, surprisingly revealing an additional lesion in the gluteal muscle on the right side (Figure 2(c)), not seen on the PET-CT (Figure 2(d)).

### 3.3. Treatment of Localized LGFMS

At the time of diagnosis 12 patients presented with localized disease. The standard treatment at the Aarhus Sarcoma Centre was surgery with an intended wide margin. The surgical margins were defined according to the principles of Enneking [7]. Margins were classsified as marginal if the incision was within the pseudocapsules, or as wide if the tumor was surrounded by a cuff of normal tissue. The surgical margin was wide in four cases and marginal in eight cases. One case was treated with adjuvant radiotherapy, 50 Gray (Gy) on 25 fractions after a marginal excision. Local recurrences, without evidence of distant metastasis, were observed in four cases. These were, like the primary tumors, treated with surgery without adjuvant therapy. 

### 3.4. Treatment of Metastatic LGFMS

Overall, three patients developed metastases. Two cases presented with metastatic disease at diagnosis, whereas one case developed metastases after removal of three consecutive local recurrences. The treatment of the metastatic LGFMS differed.

Case 1 presented with localized disease initially and developed metastases to the lung and retroperitoneum 12 years after the first treatment. Over the next 12 years the patient was treated with various types of chemotherapy including Ifosfamide, Doxorubicin, Imatinib, Trabectedin, Gemcitabine, Docetaxel, and palliative radiotherapy. During treatment, the patient developed metastases in the abdominal wall, antecubital region, and intra-abdominally and died 4 months after the last treatment, at the age of 32. The best response to chemotherapy was seen during treatment with Trabectedin. The patient was treated in two periods receiving 8 and 6 series, respectively, both with stabilization of previous progression.

Case 13 presented with multiple lung metastases with pleural involvement and a primary gluteal tumor. The patient was treated with 7 series of Doxorubicin. 19 months after finishing treatment, CT scans showed progression of the lung metastases. However, the patient did not have any symptoms and was reluctant to receive new chemotherapy; therefore it was agreed to await further development. Treatment with Trabectedin was initiated after further progression of the lung metastases as well as development of liver metastasis and a gluteal satellite tumor. After 12 series of Trabectedin, with stable disease, a CT scan showed progression and the treatment was discontinued. Treatment with antiestrogen, Tamoxifen is currently initiated. 

Case 14 was treated solely with surgery, without adjuvant radio- or chemotherapy. The patient was treated for metastases to the lungs initially, as well as 5 and 11 months after the first treatment. The primary tumor, located in the gluteal area, was discovered and removed 3 months later. Furthermore the patient was treated for metastases to the brachium at 17 and 28 months, pelvic and thigh bilaterally at 19 months, and retroperitoneum at 30 months after the first treatment. At the time of this study the patient had been disease-free for 4 months.

### 3.5. Followup

Patients were followed with clinical controls at the Sarcoma Centre between 2.5 and 24.3 years, with a median of 5.0 years. At the time of this study eight patients (57.1% (95% CI 28.9–82.3)) were disease-free and had not had any relapses. Three patients (21.4% (95% CI 4.7–50.7)) experienced only a local recurrence, after a disease-free interval of 1.9 to 17.5 years. The disease course was more complex, with distant metastasis, in the remaining three cases (21.4% (95% CI 4.7–50.7)), as described in detail in the previous section. In two out of the three patients with metastases the primary tumor was located in the gluteal area, meaning that 50% (95% CI 7–93) of patients with a gluteal tumor developed metastases. The primary tumor was located in the pelvic area in the last metastatic case.

### 3.6. *FUS* Break-Apart

Three cases (21.4% (95% CI 4.7–50.7)), cases 4, 6, and 12, were found with FISH analysis to have a break-apart in the *FUS* gene (indicating the *FUS/CREBL3L2* translocation) with 85%, 35%, and 95% rearrangement, respectively. When comparing patients who tested positive for the *FUS *break-apart with patients who tested negative, there were no difference in age (30 (95% CI 0–62) versus 37 (95% CI 23–50) years, *P* = 0.58), sex (67% (95% CI 1–91) versus 64% (95% CI 31–89) females, *P* = 0.92), tumor size (6 (range 3–26) versus 4 (range 2–25) cm, *P* = 0.51), local recurrence (33% (95% CI 1–91) versus 27% (95% CI 6–61), *P* = 0.84), and metastasis (0% (95% CI 0–71) versus 27% (95% CI 6–61), *P* = 0.31). Case 6 experienced a local recurrence after 4.1 years, whereas case 4 and 12 were disease-free after 5.0 and 3.4 years of followup, respectively. 

## 4. Discussion

The incidence of 0.18 per million reported in this study is, as expected, low. The incidence of this rare type of sarcoma has not previously been reported, probably because of the difficulty concerning the diagnosis, as well as the overlapping histological features with both benign and high-grade malignancies. Patients are often misdiagnosed with fibromatosis, neurofibroma, or malignant fibrous histiocytoma instead of LGFMS [8, 9]. In an effort to identify all cases of LGFMS in our study period, the pathological slides of all patients with a low-grade fibrosarcoma were reviewed retrospectively. Patients with aggressive fibromatosis in the ASR have been revised in relation to a previously published study [10]; however patients with certain well-defined tumors with overlapping histological features, for example, deep benign fibrous histiocytoma and nodular fasciitis, are not registered in the ASR and have therefore not been reviewed. Furthermore the ASR, which was used as the data source to identify patients with low-grade fibrosarcoma, is not a perfect reference. The completeness is 85.3% on an individual level, and 14.7% of the population were therefore not adequately queried. The incidence found in this study is therefore a conservative estimate.

The patient characteristics were consistent with the findings of other studies of LGFMS patients, with occurrences in middle-aged patients, primarily located subfascially in the proximal extremities and trunk. Our study showed that the majority (64%) of patients were females. The sex distribution differs among the published studies, and the small number of patients in each study makes these results ambiguous [2, 11–15].

The increased use of PET-CT in the determination of disease status poses a potential problem, in the case of LGFMS. The primary gluteal tumor in case 14 was PET negative on the initial scan and therefore not removed until 3 months later. Furthermore, the metastasis in the right gluteal region was only visible on the additional MRI scan. PET-CT should therefore be used with caution in patients with LGFMS, especially in cases with an unusual presentation of tumor, and perhaps a full-body MRI scan is superior in determining the dissemination of the disease. The atypical behaviour of LGFMS with a relatively high risk of metastasis also indicates that a vigorous followup schedule with imaging, for example, full-body CT or MRI, is needed.

In our study, 3 out of 14 patients (21%) developed distant metastases to nonlung anatomical locations. Generally, low-grade soft tissue sarcomas rarely metastasize, whereas high-grade sarcomas often metastasize haematogenously to the lungs [16–18]. Metastases to lymph nodes or other anatomical locations are generally rare, except for a few well-defined subgroups with atypical patterns of metastasizing, for example, myxoid liposarcoma [19, 20]. Previous studies report that the rate of metastases in LGFMS varied from 5% to 41%; however it is likely that the studies reporting the highest proportions overestimated the metastatic rate, by mostly including patients with unexplained metastasis [2, 11, 21].

Interestingly, all of the patients with metastatic disease had their primary tumor located in the lower trunk area (gluteal/pelvic). Due to the small number of patients, this can merely be coincidental, and when reviewing the literature there was no clear tendency towards tumors located in this area being associated with a more aggressive course;d however it is worth noticing.

The treatment of these low-grade tumors with high metastatic potential is difficult. The “traditional” approach to low-grade sarcomas is surgical removal, without adjuvant therapy, since the recurrence, if any, is usually local. However in the cases presented in this study, this approach is inadequate or impossible due to the multiple lesions. Due to the low grade of malignancy and therefore the low mitotic rate, LGFMS is not expected to be very chemo- or radiosensitive. The best response to chemotherapy in our study was short-term stabilization of disease progression, seen with Trabectedin. However the lack of tumor response may indicate that this was due to the natural history of the tumor itself rather than any effect of the drug. A search of the existing literature revealed no treatment policy regarding the use of chemo- and radiotherapy, and the current knowledge and experience on how to treat patients with metastatic LGFMS are scant. A recent review suggested that Trabectedin could be particularly effective in translocation-related soft tissue sarcomas [22]. Since LGFMS can be considered related to the *FUS/GREB3L2* fusion gene, the use of Trabectedin may offer some benefit. Unfortunately the two cases treated with chemotherapy in our study were negative for the *FUS* break-apart.

Of the three cases with metastatic LFGMS in our study, one is currently disease-free. At present, surgery is the only treatment that results in disease-free periods; however, further investigation into the management of metastatic low-grade tumors is necessary, and in this context, a discussion of considering “no treatment” as a strategy is crucial. 

Three cases (21.4%) were found to be positive for the *FUS *break-apart in our study. The proportion of LGFMS cases positive for the *FUS *break-apart reported in the existing literature varies considerably. Consistent with our findings, Panagopoulos et al. [13] reported 12 positive cases out of 59 LGFMS patients; however, other studies have shown proportions of positive cases ranging from 81.1% to 96% [12, 14, 15]. The large variation in the proportion of positive cases in different studies might be explained by differences in local practices and inclusion criteria. While the diagnosis of LGFMS “traditionally” is made based on the characteristic histological appearance, some centres use the *FUS *break-apart analysis as a part of the diagnostics, resulting in a, not surprisingly, high proportion of positive cases. Corresponding to the results other studies have reported, there was no significant difference in age, sex, tumor size, local recurrence, or metastasis when comparing patients who tested positive for the *FUS *break-apart with patients who tested negative [14]. It should, however, be noted that the 0% in the *FUS* break-apart positive group developed metastases compared with 27% in the *FUS* break-apart negative group, and thus it can be questioned if there truly is a difference that cannot be detected due to the small sample size.

## 5. Conclusions

In conclusion LGFMS is a rare disease with multiple challenges, as described in our cases: diagnosing LGFMS, and determining disease status, as well as the treatment strategy in cases with metastatic disease. No apparent responses to chemotherapy were seen, and at present, surgery is the only treatment that results in disease-free periods. The prevalence of the *FUS *break-apart in our study was 21.4%. We found no significant difference in local recurrence or metastasis in proportion to the *FUS *break-apart. To date, our knowledge of LGFMS is still sparse, and further research in the nature of the disease is essential to diagnose and treat LGFMS optimally.

## Figures and Tables

**Table 1:** Clinical data, followup, and *FUS *break-apart in 14 patients with low-grade fibromyxoid sarcoma, diagnosed at the Sarcoma Centre of Aarhus University Hospital in the period of 1979 to 2010.

Case	Age/sex	Size	Location	Depth	Treatment	Margin	Relapse time/location	Relapse treatment	Followup	*FUS *
1	8/M	8	Pelvic	Subfascial	Surgery	Marginal	8.0/Local, intra-abdominal	Surgery, chemo- + radiotherapy	24.3	Neg.
2	38/F	4	Hand	Subfascial	Surgery + radiotherapy	Marginal	17.5/Local	Surgery	21.7	Neg.
3	54/F	3	Thigh	Subcutaneous	Surgery	Wide			6.0	Neg.
4	37/F	6	Thigh	Subfascial	Surgery	Wide			5.0	Pos.
5	14/M	5	Brachium	Subfascial	Surgery	Marginal			4.8	Neg.
6	37/F	26	Gluteal	Subfascial	Surgery	Marginal	4.1/Local	None	9.0	Pos.
7	53/F	2	Neck/head	Subfascial	Surgery	Marginal			7.7	Neg.
8	24/F	25	Abdominal wall	Subfascial	Surgery	Wide			4.6	Neg.
9	30/F	3	Abdominal wall	Subfascial	Surgery	Marginal	1.9/Local	Surgery	2.5	Neg.
10	35/M	4	Neck/head	Subfascial	Surgery	Wide			5.2	Neg.
11	64/M	9	Gluteal	Subfascial	Surgery	Marginal			5.0	Neg.
12	14/M	3	Brachium	Subfascial	Surgery	Marginal			4.0	Pos.
13	18/F	9	Gluteal	Subfascial	Chemotherapy		0/Lung, liver	Chemotherapy	3.2	Neg.
14	63/F	3	Gluteal	Subfascial	Surgery	Marginal	−0.3/Lung, brachium, and thigh	Surgery	2.8	Neg.

## References

[1] Evans HL (1987). Low-grade fibromyxoid sarcoma. A report of two metastasizing neoplasms having a deceptively benign appearance. *American Journal of Clinical Pathology*.

[2] Evans HL (1993). Low-grade fibromyxoid sarcoma: a report of 12 cases. *American Journal of Surgical Pathology*.

[3] Storlazzi CT, Mertens F, Nascimento A (2003). Fusion of the FUS and BBF2H7 genes in low grade fibromyxoid sarcoma. *Human Molecular Genetics*.

[4] Reid R, De Silva MVC, Paterson L, Ryan E, Fisher C (2003). Low-grade fibromyxoid sarcoma and hyalinizing spindle cell tumor with giant rosettes share a common T(7;16)(q34;p11) translocation. *American Journal of Surgical Pathology*.

[5] Maretty-Nielsen K, Aggerholm-Pedersen N, Keller J, Safwat A, Baerentzen S, Pedersen AB (2013). Population-based Aarhus Sarcoma Registry: validity, completeness of registration, and incidence of bone and soft tissue sarcomas in western Denmark. *Clinical Epidemiology*.

[6] Statistics Denmark (2013). *Population Denmark*.

[7] Enneking WF, Spanier SS, Goodman M (1980). A system for the surgical staging of musculoskeletal sarcoma. *Clinical Orthopaedics and Related Research*.

[8] Lindberg GM, Maitra A, Gokaslan ST, Saboorian MH, Albores-Saavedra J (1999). Low grade fibromyxoid sarcoma: fine-needle aspiration cytology with histologic, cytogenetic, immunohistochemical, and ultrastructural correlation. *Cancer*.

[9] Domanski HA, Mertens F, Panagopoulos I, Åkerman M (2009). Low-grade fibromyxoid sarcoma is difficult to diagnose by fine needle aspiration cytology: a cytomorphological study of eight cases. *Cytopathology*.

[10] Sørensen A, Keller J, Nielsen OS, Jensen OM (2002). Treatment of aggressive fibromatosis: a retrospective study of 72 patients followed for 1-27 years. *Acta Orthopaedica Scandinavica*.

[11] Folpe AL, Lane KL, Paull G, Weiss SW (2000). Low-grade fibromyxoid sarcoma and hyalinizing spindle cell tumor with giant rosettes: a clinicopathologic study of 73 cases supporting their identity and assessing the impact of high-grade areas. *American Journal of Surgical Pathology*.

[12] Mertens F, Fletcher CDM, Antonescu CR (2005). Clinicopathologic and molecular genetic characterization of low-grade fibromyxoid sarcoma, and cloning of a novel FUS/CREB3L1 fusion gene. *Laboratory Investigation*.

[13] Panagopoulos I, Storlazzi CT, Fletcher CDM (2004). The chimeric FUS/CREB3L2 gene is specific for low-grade fibromyxoid sarcoma. *Genes Chromosomes and Cancer*.

[14] Rose B, Tamvakopoulos GS, Dulay K (2011). The clinical significance of the FUS-CREB3L2 translocation in low-grade fibromyxoid sarcoma. *Journal of Orthopaedic Surgery and Research*.

[15] Matsuyama A, Hisaoka M, Shimajiri S (2006). Molecular detection of FUS-CREB3L2 fusion transcripts in low-grade fibromyxoid sarcoma using formalin-fixed, paraffin-embedded tissue specimens. *American Journal of Surgical Pathology*.

[16] Pisters PWT, Leung DHY, Woodruff J, Shi W, Brennan MF (1996). Analysis of prognostic factors in 1,041 patients with localized soft tissue sarcomas of the extremities. *Journal of Clinical Oncology*.

[17] Zagars GK, Ballo MT, Pisters PWT (2003). Prognostic factors for patients with localized soft-tissue sarcoma treated with conservation surgery and radiation therapy: an analysis of 1225 patients. *Cancer*.

[18] Gustafson P (1994). Soft tisue sarcoma: epidemiology and prognosis in 508 patients. *Acta Orthopaedica Scandinavica*.

[19] Estourgie SH, Nielsen GP, Ott MJ (2002). Metastatic patterns of extremity myxoid liposarcoma and their outcome. *Journal of Surgical Oncology*.

[20] Schwab JH, Boland P, Guo T (2007). Skeletal metastases in myxoid liposarcoma: an unusual pattern of distant spread. *Annals of Surgical Oncology*.

[21] Goodlbd JR, Mentzel T, Fletcher CDM (1995). Low grade fibromyxoid sarcoma: clinicopathological analysis of eleven new cases in support of a distinct entity. *Histopathology*.

[22] Cesne AL, Cresta S, Maki RG (2012). A retrospective analysis of antitumour activity with trabectedin in translocation-related sarcomas. *European Journal of Cancer*.

